# Supplementary material for: MetaRibo-Seq measures translation in microbiomes
Source: Nat Commun. 2020 Jun 29;11:3268. doi: 10.1038/s41467-020-17081-z (PMC7324362; doi:10.1038/s41467-020-17081-z)
Supplement: Supplementary file 10 — Supplementary Data 7 [file 41467_2020_17081_MOESM10_ESM.zip › File2/Confidence_High_Taxonomy/113704_out.krona.html]

Javascript must be enabled to view this page.

members
magnitude
magnitudeUnassigned
count
unassigned
taxon
rank

113704\_out

13

13
2
superkingdom

13
976
phylum

13
200643
class

order
171549
13

family
2005525
13


SRS020869\_contig\_number\_33513
genus
375288
1
13

46503

SRS017433\_contig\_number\_10713SRS018888\_contig\_number\_7758SRS022524\_contig\_number\_9149SRS050026\_contig\_number\_8288SRS055966\_contig\_number\_41SRS063040\_contig\_number\_19690SRS076804\_contig\_number\_14377SRS077086\_contig\_number\_9462SRS104311\_contig\_number\_15321SRS140492\_contig\_number\_20071SRS140513\_contig\_number\_14814SRS149784\_contig\_number\_23626
species
12
